# Adaptation of Research Project Requirement at Pharmacy Undergraduate Studies: Students' Perception, Attitude, and Experiences

**DOI:** 10.1155/2024/8144325

**Published:** 2024-04-02

**Authors:** Najia Rahim, Kiran Rafiq, Shagufta Nesar, Sadaf Naeem, Fakhsheena Anjum, Muhammad Azhar Mughal

**Affiliations:** ^1^Pharmacy Practice, Dow College of Pharmacy, Dow University of Health Sciences, Karachi, Pakistan; ^2^Pharmaceutical Chemistry, Institute of Pharmaceutical Sciences, Jinnah Sindh Medical University, Karachi, Pakistan; ^3^Jinnah College of Pharmacy, Sohail University, Karachi, Pakistan; ^4^Pharmacy Practice, Institute of Pharmaceutical Sciences, Jinnah Sindh Medical University, Karachi, Pakistan; ^5^Pharmacology and Therapeutics, Faculty of Basic Medical Sciences, Jinnah Sindh Medical University, Karachi, Pakistan

## Abstract

**Objective:**

To determine the final year pharmacy undergraduate students' attitudes toward research after completing a research project.

**Methods:**

A research project was introduced in the final year of the PharmD program in January 2022. After a period of one year, in Janurary 2023, students submitted their final research to the faculty members. The survey was conducted from 1st March to 30st April 2023 using a study tool that contained items asking students' demographic, their research perceptions, attitude and experience, and also motivation/barriers faced during the research project. Descriptive and *t*-test statistics were utilized to compare the means of subgroups at a level of significance, i.e., *p* < 0.05. The data were also analyzed using Goodman and Kruskal's gamma and Mann–Whitney *U* test.

**Results:**

Majority of the students (93.8%) agreed regarding the significance of research in the pharmacy profession. Students were found to have their projects a worthwhile learning opportunity (94.2%). Students' motivation to execute research project stems from mandatory curriculum courses, improving clinical or hospital pharmacist training and fulfilling research skills (90%). Barriers hindered include lack of training, time, and patient follow-up (approximately 70%).

**Conclusion:**

The current study's finding was concluded with the fact that research is a valuable component of a well-rounded education and can enhance a pharmacist's skills. However, they need a combination of formal education and practical experience to pursue a profession in pharmacy.

## 1. Introduction

Research is regarded as a crucial part of healthcare education for its emphasis on demonstrating the reliability of information, which enables healthcare professionals to make wise judgments. Research impacts the diagnosis, prevention, and treatment of illness in a positive direction [1]. Pharmaceutical research focused on evidence-based information is the key to improve the healthcare system. Drug therapy has interdisciplinary aspects, which increases the need for pharmaceutical research to optimize new services, inform policies, and lead to real change in pharmacotherapy [2, 3].

Undergraduate studies require increased exposure to evidence-based pharmacy practice for the improvement of baseline knowledge of pharmaceutical care resulting in graduate students' research productivity [4]. Furthermore, research also supports students to advance their skills of critical thinking and problem-solving obligatory as a pharmacist to interpret, use, and communicate the results of research effectively. These skills can be improved through knowledge building and disseminated through research activities in pharmacy graduate studies for further development of the pharmacy profession globally [5, 6]. In America, pharmacy education was revolutionized when the Accreditation Council for Pharmacy Education released a document that strongly recommended the integration of research into Doctor of Pharmacy (PharmD) curricula [7]. However, there is a mixed response from different pharmacy educational institutes to the research requirement in undergraduate studies [8].

Pharmaceutical care, evidence-based practice, and quality assurance turn out to be the core of the pharmacy profession. Pharmacy educators have to train pharmacy students in such a way that they can meet the extended challenges of their profession [9]. The Drug Regulatory Authority of Pakistan, along with other professional bodies like Pakistan Pharmacists Association (PPA), should work together to develop a well-structured advanced healthcare system in Pakistan where pharmacists can play their defined role. It is very inspiring that Pakistani pharmacy students were inclined towards practicing pharmaceutical care [10, 11]. In Pakistan, PharmD program was introduced back in 2004 [12]; however, the pharmacy-practice department focusing on clinical pharmacy was first implemented in 2014 [13]. Clerkship in a clinical setting and submitting a report related to the clinical pharmacy-practice course completed by the students and evaluated by the external examiner are part of the revised PharmD curriculum as approved by Pakistan Pharmacy Council and Higher Education Commission of Pakistan [14]. The establishment of pharmacy services in hospitals is indeed for the success of this research-oriented amendment in curriculum.

Students during their undergraduate studies are more willing to complete their graduation instead of engaging in any research activity. Understanding these attitudes is necessary to help instructors craft more positive attitudes towards courses involving research. In our investigation of the literature, we were unable to find any published work demonstrating the research-related experiences of pharmacy undergraduate students from Pakistan after completing a research requirement in their final year. Therefore, the aim of this study is to introduce a research project in their graduate program and after completing the research ask for their responses regarding their perceptions, attitudes, and experiences concerning research.

## 2. Methodology

### 2.1. Study Design and Setting

A research project was introduced in the final year of the PharmD program to increase students' inclination towards research in January 2022. After a period of one year, in Janurary 2023, students submitted their final research to the faculty members. It was necessary to evaluate the success of this newly introduced research requirement. A cross-sectional survey-based study was executed. Survey was conducted from 1^st^ March 2023 to 30^st^ April 2023 after submission of research projects using a study tool among students from pharmacy colleges in Karachi.

### 2.2. Study Population

Survey was conducted among students from two colleges in Karachi, where the research project requirement was implemented. A total of ten colleges and institutes are offering PharmD in Karachi; however, only two colleges took initiative to introduce the research activity during their undergraduate study. Therefore, students belonging to these colleges were surveyed.

### 2.3. Sample Size and Sampling

Raosoft sample size calculator (Raosoft Inc.®, Seattle, DC, USA) was used to calculate the sample size by utilizing a confidence level of 99% with a 5% margin error (https://www.raosoft.com/samplesize.html, accessed on 28^th^ September 2022). The sample size was 182. However, 200 questionnaires were distributed to cater the nonresponse issue. Nonprobability sampling technique was used.

### 2.4. Inclusion/Exclusion Criteria

Inclusion criteria were the undergraduate final-year pharmacy students of two pharmacy colleges in the metropolitan city of Karachi involved in research during their undergraduate program. Undergraduate students who were in their first to fourth year of PharmD and not involved in any research activity were excluded from the study.

### 2.5. Data Collection Tool and Methods

For this study, a study tool was adopted from previous studies [15, 16] and transformed. The tool's initial draft contained items to investigate students' perceptions of research initiatives. The questionnaire was reviewed by a panel of ten experts, and ten items were deleted from the initial draft. Revalidation was done on the first draft's content. Ten pharmacy experts who were active in research and practice made up the expert group. The panel evaluated the study tool's validity. The content validity index (CVI) and content validity ratio (CVR) were calculated by asking every expert to mark each item in the questionnaire as 1 = essential or 0 = nonessential from the perspective of a pharmacy student [17]. The final validated draft of the tool had a total of fifteen items. All items were responded to on a five-Likert scale, i.e., strongly agree = 5, agree = 4, neutral = 3, disagree = 2, and strongly disagree = 1. Strongly agree and agree were considered positive responses. Strongly disagree and disagree were negative responses. Reliability was assessed using the Cronbach Alpha method. Internal consistency, i.e., correlation coefficient with Pearson through a 2-tailed level of significance, was also judged at a level of significance of 0.05 [18]. Respondents were stimulated to complete the surveys provided within the prescribed time period of 15 minutes. Responses were gathered and evaluated. Investigator obtained approval from the head of each institution before starting the study.

### 2.6. Data Analysis

All data were analyzed through IBM SPSS version 22, Armonk, NY, USA. The demographic data were reported in sample counts (*N*) and percentages (%). Statistically significant association was determined by applying the *t*-test to compare the means of two independent samples of gender and institutions. Statistically significant association was also determined by nonparametric tests, including the Mann–Whitney *U* test and Goodman and Kruskal's gamma test. Statistical significance was considered at a *p* value less than 0.05.

### 2.7. Ethics Approval and Informed Consent

The participants were explained about the objectives of the study, and their written consent was sought earlier taking their responses. They were also guaranteed of the confidentiality of their personal information and responses. The study was approved by the Ethical Review Committee of Sohail University, Karachi (Protocol# 252/23).

## 3. Results

### 3.1. Questionnaire Validation

The study tool was revalidated for content and found to be validated with I-CVIave (0.866) and S-CVIave (0.859). Internal reliability was also observed. Cronbach alpha was found to be 0.892. Item-item correlation ranged between 0.118 and 0.576 except for one correlation between item #4 and item #9, i.e., 0.671, which was ignored. Interitem correlation values between 0.15 and 0.50 show a good correlation. A value greater than 0.50 (i.e., 0.671) means that items are correlated to a greater extent and may be repetitive in calculating the intended construct. Internal consistency, i.e., correlation coefficient with Pearson through a 2-tailed level of significance, revealed that item #1 and item#14 correlation were not significantly correlated with *p*=0.104; however, all other items of the survey were significantly correlated (*p* < 0.05).

### 3.2. Survey Results

A total of 191 students responded (response rate was 76.4%). Their demographics are mentioned in Table 1. Pharmacy undergraduate students thought that research is important, especially during their PharmD program as their degree is more pharmacy-practice-oriented. Their mean score for different items of the study tool ranged between 3.86 and 4.71 (SD ranged from 0.622 to 0.928). Students replied positively to all items as they were inclined towards research during and after completing their graduation (strongly agree and agree percentages were between 73.4% and 98.5%).

**Figure 1:**
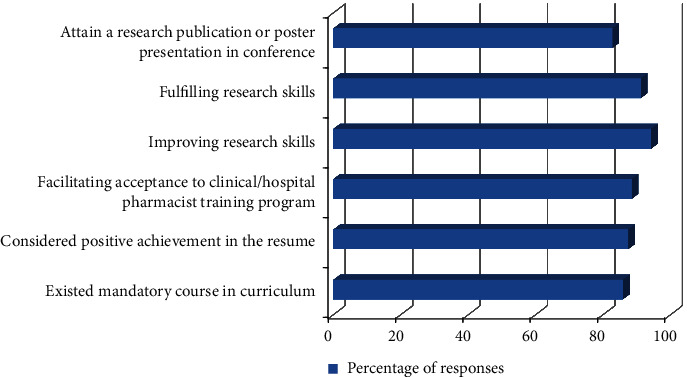
Student's responses regarding motivation felt during their research project acquisition and submission.

**Figure 2:**
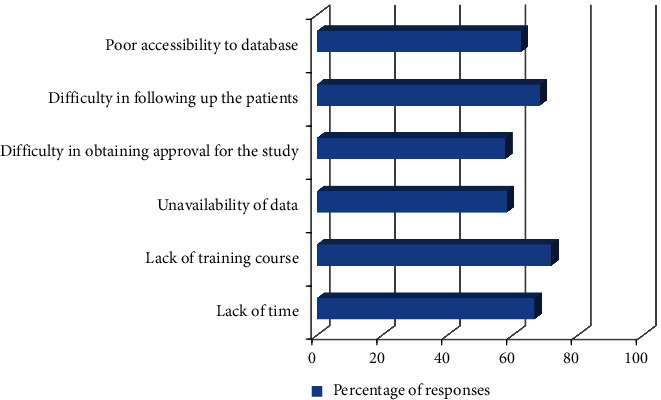
Student's responses regarding barriers faced during their research project acquisition and submission.

Students were also interrogated regarding motivation and barriers faced during this research project's acquisition and submission. Their responses are shown in Figures 1 and 2. Most of the students showed motivation for the research due to fulfilling and improving their research skills (90%). The barrier they faced during research was a lack of time and training, as well as difficulty following up with the patient during their research (approximately 70%).

### 3.3. Statistical Analysis of Students' Response

A statistically significant association was determined by applying the *t*-test to compare the means of two independent samples of gender and institutions mentioned in Table 2. There was a significant difference in the responses of males and females (*p*=0.044, 95% CI = 0.010–0.700) as well as students of both institutions (*p*=0.009, 95% CI = 0.082–0.572) to the question, i.e., “Overall, I was pleased with my selection of an advisor for my project.” As both are independent institutions, students' responses to many questions in the survey were significantly different using the level of significance 0.05, i.e., “Overall, the project was a valuable learning experience” (*p*=0.008, 95% CI = 0.063–0.416) and “By the end of the process, I felt that I had acquired enough knowledge and skills that I could independently conduct a similar research project” (*p*=0.010, 95% CI = 0.078–0.569).

A statistically significant association was also determined by nonparametric tests, including Goodman and Kruskal's gamma test and Mann–Whitney *U* test using *p* < 0.05. Results are mentioned in Table 3. Goodman and Kruskal's gamma test only suggests correlation, whether strong or weak. *p* < 0.05 means a statistically significant association but not very strong as the *G* value is less than 0.5, which we consider a strong correlation. Goodman and Kruskal's gamma test revealed a moderately negative correlation among the students from two institutions, where survey was conducted in response to some questions, i.e., “Overall, I was pleased with my selection of an advisor for my project” (*G* = −0.347, *p* = 0.021) and “Overall, the project was a valuable learning experience” (*G* = −0.366, *p* = 0.005). Institution also influences the response to the question “The School of Pharmacy should continue to require a research project in the pharmaceutical care pathway” (*G* = −0.333, *p* = 0.01). The Mann–Whitney *U* test revealed that there was no significant association between gender and their response except for one item in which students responded regarding their selection of advisor (*p* = 0.03).

## 4. Discussion

Students, after high school education, enter the graduate system and meet a new culture full of struggle, self-study, and challenging projects. Along the way, they explore themselves for their coping abilities. That practice makes them aware of professional demands and prepares them for future organizational behavior [19]. Meanwhile, the research becomes an essential component of the curriculum during their graduation, enhancing their learning capabilities to become junior researchers for future research [20]. Although beliefs and preferences influence research interests and the value of research, students were more likely to wish to conduct research when a mentor was accessible, even though many thought they lacked the competence and self-assurance to accomplish their research [21].

According to the available data, the current study was intended to examine the degree of acceptance for research projects among undergraduate students and aimed at investigating the facilities offered and outcomes among pharmacy undergraduate students. The two pharmacy graduating institutes were selected as the target population, which was of both genders. The study's findings were amazing because it was determined that the importance of the research among students was high. However, some do not agree on the importance of research. There may be an assortment of reasons for this, including a lack of training courses, insufficient resources, and lack of time owing to laboring for pay or other commitments. Another researcher also reported undergraduate students' perceived barrier regarding their research including unawareness of research activities and their benefits; unable to work independently; difficulty in making contact with senior faculty, and so on [22]. Unfortunately, the outputs of the current data are not considered sufficient regarding the goodwill of the study project's participating students, and it is highly uncertain whether or not this additional research requirement will assist them professionally. To remove this ambiguity, students who have entered the professional career would be asked about the expediency of such research activities in their career. Much more work would have been done previously to support the value of these research activities as an ideal model for providing baseline knowledge and skills for research in their professional careers, but the evidence was weak [22, 23].

The majority of the respondents (83%) had excellent research heads, and everyone expressed their high levels of satisfaction with their research heads. However, a statistically significant difference (*p* < 0.05) was observed among students belonging to different institutes; however, institute names are kept anonymous (Table 3). This demonstrates that the faculty members who are currently available are highly competent, experts in their fields, and supportive of researchers. According to a scholar, the primary goal of any researcher, whether they are faculty members or aspiring undergraduates, is to master a method of question-asking and give it a shot [24]. Another question concerned the topic choice for the project, and the results were favorable as most researchers found the subject to be imperative and of node value. The selection of the theme for undergraduates can be based on either the choice of the supervisor or researcher or according to the desire of either of them. Mostly, the supervisor gives the idea of the theme to the students, and the wise minds make it more attractive and novel through their modern approaches, as, nowadays, artificial intelligence has given a diversified explosion to the young generation [25]. Similarly, many undergraduates are busy with content writing and medical transcription, which also help them search for the most current and innovative topics. It is necessary to build a two-way consensus on research topic selection to get a positive response from students, as observed in this study. As there is an experience difference between the two, the young researcher and supervisor have different visions, high ideas, and move fast without considering the pros and cons, whereas the experienced mentor keeps an eye out and has in-depth knowledge about all the obstacles and peripheries [21, 23].

Universities are the hub of research, and the faculty under their umbrella remains engaged in research at all times despite having hectic teaching responsibilities and projects. The rigorous struggle strengthens their hidden eagerness for research and mentorship, which is not only pivotal for career growth but also essential for driving modernization and the global recognition of universities. Good mentoring is proven by the time and devotion of the supervisor. The present study showed very strong satisfaction regarding selection and trust in the supervisor or mentor from students. With each of their students, good mentors spend an immense amount of time talking about science, teaching them how to design efficient experiments, interpret and analyze data, create research articles and grants, review manuscripts for journals, practice lectures, and offer career advice. They encourage students to participate in activities that would help in research work, like statistics, Python, block chain, simulation, computational technique courses, and attending conferences, seminars, poster presentations, and so on [22, 25]. However, a previous report documented that faculty member who guides fewer students research projects can contribute more positively in student learning experience than those faculty members who provided guidance to more number of projects [16].

During the present study, students significantly agreed with the fact that research is valuable learning for them because engaging in research as part of their academic curriculum opens numerous opportunities and gains for personal and scholarly intensification. As research enables students to apply theoretical concepts to real-world situations, improving their understanding and critical thinking skills, it helps them identify problems, formulate questions, and design methodologies, developing analytical and problem-solving abilities. Research projects require independent work, self-discipline, and responsibility. It introduces students to various methodologies, data collection techniques, and data analysis, enhancing their communication and conflict resolution skills. Research also provides students with firsthand experience in applying the scientific method, leading to publications, academic profile boosts, and opportunities for networking with professors, researchers, and professionals. Universities support undergraduate research through programs, initiatives, and funding opportunities [26]. A slight difference was observed between the feelings of students before starting and at the completion of research, as the first one was established as nonsignificant; however, later on, the thought process was observed to be statistically significant after achieving the goal, which shows the satisfaction of students from overall experience. A study conducted in USA and Finland reported that the students from USA were confused whether they will need research skills in their future work and faced added difficulties in research courses; however, Finland students responded a slightly more positive view after completing their research-oriented course [27].

In a query regarding satisfaction about the outcome of research, the students showed satisfactory results, and the outcomes established include various factors, most prominently the sense of accomplishment and achievement that come from witnessing the results of one's hard work. Furthermore, if the research topic reflects the student's interests and ardors, the journey of research would be more pleasurable and momentous [28]. Research involves both the learning process and the final product. Saudi pharmacy students also believed that they have learned useful skills, increased their knowledge, and developed intellectually and found to be satisfied with their research experience [29]. Increased pleasure can be attributed to productive peer collaboration, strong mentor-student relationships, and important research findings. Having research experiences that match a student's career goals, overcoming obstacles, and giving good feedback can all increase satisfaction. By offering assistance, resources, and chances for advancement, institutions and mentors can play a significant role in encouraging a pleasant research experience.

The response was found to be agreeable with a nonsignificant difference among subgroups for the question asked to the students about perusing the projects in the future. It has been documented that pharmacy USA graduates who completed research projects during graduation continued to do at least one project since graduation resulting in poster/paper presentations [30]. There are many reasons for showing such established facts. The most likely are attaining and improving research skills and making them acceptable for the pharmacy profession. Another study reported that female pharmacy students choose a research project in the field of clinical pharmacy as in general most of the pharmacy students plan to work in hospitals after completing graduation [31]. However, it is essential to discuss the important ground reality that undergraduate research does not pay off well in a career, specifically for those pharmacists who opt for marketing, business development, and sales. Hence, keeping reality in mind, the projects may be designed accordingly to accomplish the demands and prepare smart and diversified professionals. Relating the fact, responses were ascertained to be significant in favor of designing such research projects that could be relevant to the pharmaceutical market demand and could support the future of pharmacists in all aspects [6]. The pharmacy students showed more or less similar, statistically nonsignificant responses for doing research, due to uncertain position of pharmacists in business, especially in corporate organizations where business diplomas and degrees could support them better, as the research approaches in pharmacy careers are not enough to strengthen the career and hence do not impact the market value of a pharmacist. According to some previous reports, the incorporation of research projects into the curriculum has proven useless in distinguishing them from other universities that do not undertake research. However, the importance of research in the health care profession is a recognized datum. These research activities would be a foundation stone for building a pharmacist to meet the twenty-first century prerequisites of the pharmacy profession.

These types of studies to ascertain Pakistani students' perceptions and attitudes towards research are extremely rare. To establish research-training program in universities during undergraduate studies, their answers and experiences provide guidance to the institutions, accrediting authorities, and government. The fact that we only received replies from Karachi-based institutes limits the generalizability of the study's findings. There are five provinces in Pakistan, having a large number of pharmacy institutes. Students belonging to different regions of Pakistan might have diverse attitudes regarding research, as there are differences in research environment as well as resources available in their institutes. The study's small sample size was also a shortcoming, because it did not include students, who were not involved in research during all five academic years. Students studying in such institutes, where research project has not been implemented, might have different perception. We conducted a survey among students within three months of completing the research assignment. Longitudinal assessments conducted after two-five years of their graduation are recommended for more accurate assessment of the impact of research activity on the graduates' capacity to carry out comparable research, while in practice. The study's preliminary findings should be used with caution.

## 5. Conclusion

Students showed inclination towards research during and after completing their graduation. The research activity developed analytical and problem-solving abilities in them. The present study concludes that research is a precious element of a well-formed education and can augment a pharmacist's skills, but it is not the exclusive pathway to becoming a diversified and viable pharmacist. To pursue a successful career in pharmacy, one must possess a combination of formal education, licensure, and real-world experience.

## Figures and Tables

**Table 1:** Demographics of study population.

Study population characteristics		
Age	Mean ± SD	23.62 ± 1.13

Gender	Female″	163 (84.9)
	Male	28 (14.6)

Institutes	Institute#1″	109 (56.8)
	Institute#2	82 (42.7)

″Values mentioned as numbers (percentage).

**Table 2:** Descriptive statistics of responses received from study population with results of *t*-test at *p* < 0.05.

Items	Mean	Standard deviation	Gender (*p* value)	Institution (*p* value)
Research is important	4.71	0.479	0.446	0.551
Research conducting during PharmD is important	4.59	0.554	0.602	0.109
Conducting this research project was valuable for me professionally	4.40	0.673	0.696	0.125
Overall, I was pleased with my selection of a topic for my project	4.13	0.750	0.896	0.303
Overall, I was pleased with my selection of an advisor for my project	4.13	0.861	0.044^*∗*^	0.009^*∗*^
Overall, the project was a valuable learning experience	4.32	0.622	0.729	0.008^*∗*^
At the start of the process, I felt that I was adequately prepared to take on my project	3.86	0.860	0.847	0.978
By the end of the process, I felt that I had acquired enough knowledge and skills that I could independently conduct a similar research project	3.99	0.864	0.687	0.010^*∗*^
I am pleased with the outcome/results of my project I am (or will be) exploring	3.99	0.729	0.811	0.608
Because of my project, I am more likely to conduct research in the future	3.99	0.771	0.822	0.403
The amount of time I was able to spend on my project was adequate	3.87	0.928	0.213	0.254
The school of pharmacy should continue to require a research project in the pharmaceutical care pathway	4.43	0.645	0.712	0.029^*∗*^
As I conducted a project, I am more qualified marketable	3.96	0.800	0.287	0.794
The research project distinguishes me from PharmD students at other schools	3.99	0.811	0.195	0.412
I feel research is an important part of the profession of pharmacy	4.42	0.635	0.968	0.524

^*∗*^Statistically significant association.

**Table 3:** Statistically significant association of responses received from study population with their gender and institution (results of Mann–Whitney *U* and goodman and Kruskal's gamma test at *p* < 0.05).

Items	Mann–Whitney *U* test		Goodman and Kruskal's gamma test	
	Gender	Institution	Gender	Institution
Research is important	0.371	0.411	0.189 (0.393)	−0.130 (0.412)
Research conducting during PharmD is important	0.431	0.064	0.156 (0.428)	−0.261 (0.063)
Conducting this research project was valuable for me professionally	0.423	0.120	0.151 (0.396)	−0.205 (0.117)
Overall, I was pleased with my selection of a topic for my project	0.743	0.276	−0.059 (0.736)	−0.138 (0.268)
Overall, I was pleased with my selection of an advisor for my project	0.030^*∗*^	0.004^*∗*^	−0.385 (0.021)^*∗*^	−0.347 (0.003)^*∗*^
Overall, the project was a valuable learning experience	0.857	0.007^*∗*^	−0.035 (0.854)	−0.366 (0.005)^*∗*^
At the start of the process, I felt that I was adequately prepared to take on my project	0.699	0.916	−0.067 (0.692)	−0.013 (0.915)
By the end of the process, I felt that I had acquired enough knowledge and skills that I could independently conduct a similar research project	0.750	0.009^*∗*^	0.055 (0.749)	−0.315 (0.008)^*∗*^
I am pleased with the outcome/results of my project I am (or will be) exploring	0.920	0.584	0.018 (0.922)	−0.073 (0.562)
Because of my project, I am more likely to conduct research in the future	0.759	0.633	0.052 (0.769)	0.055 (0.658)
The amount of time I was able to spend on my project was adequate	0.183	0.115	−0.224 (0.200)	0.193 (0.113)
The school of pharmacy should continue to require a research project in the pharmaceutical care pathway	0.622	0.011^*∗*^	0.093 (0.622)	−0.333 (0.010)^*∗*^
As I conducted a project, I am more qualified marketable	0.286	0.787	−0.100 (0.227)	0.033 (0.785)
The research project distinguishes me from PharmD students at other schools	0.191	0.486	−0.223 (0.171)	−0.087 (0.481)
I feel research is an important part of the profession of pharmacy	0.869	0.424	0.032 (0.865)	−0.108 (0.421)

^*∗*^Statistically significant association.

## Data Availability

Data supporting the findings of this study are available from corresponding author upon reasonable request.
